# Trends in Adherence to Recommended Cancer Screening: The US Population and Working Cancer Survivors

**DOI:** 10.3389/fonc.2012.00190

**Published:** 2012-12-27

**Authors:** Tainya C. Clarke, Hosanna Soler-Vila, Lora E. Fleming, Sharon L. Christ, David J. Lee, Kristopher L. Arheart

**Affiliations:** ^1^Department of Epidemiology and Public Health, Miller School of Medicine, University of MiamiMiami, FL, USA; ^2^Department of Analytical Chemistry and Instrumental Analysis, Autónoma University of MadridMadrid, Spain; ^3^European Centre for Environment and Human Health, Peninsula College of Medicine and DentistryTruro, UK; ^4^Department of Human Development and Family Studies, Purdue UniversityWest Lafayette, IN, USA; ^5^Department of Statistics, Purdue UniversityWest Lafayette, IN, USA

**Keywords:** cancer, screening, survivors, occupation, mammogram, papanicolaou test, colorectal screening

## Abstract

**Introduction**: Over the past decade the United States (US) has seen a decrease in advanced cancer diagnoses. There has also been an increase in the number of cancer survivors returning to work. Cancer screening behaviors among survivors may play an important role in their return-to-work process. Adherence to a post-treatment cancer screening protocol increases early detection of secondary tumors and reduces potentially limiting side-effects. We compared screening trends among all cancer survivors, working survivors, and the general population over the last decade. **Materials and Methods**: Trends in adherence to recommended screening were analyzed by site-specific cancer. We used the Healthy People goals as a measure of desired adherence. We selected participants 18+ years from 1997 to 2010 National Health Interview Survey for years where detailed cancer screening information was available. Using the recommendations of the American Cancer Society as a guide, we assessed adherence to cancer screening across the decade. There were 174,393 participants. Analyses included 7,528 working cancer survivors representing 3.8 million US workers, and 119,374 adults representing more than 100 million working Americans with no cancer history. **Results**: The US population met the Healthy People 2010 goal for colorectal screening, but declined in all other recommended cancer screening. Cancer survivors met and maintained the HP2010 goal for all, except cervical cancer screening. Survivors had higher screening rates than the general population. Among survivors, white-collar and service occupations had higher screening rates than blue-collar survivors. **Conclusion**: Cancer survivors report higher screening rates than the general population. Nevertheless, national screening rates are lower than desired, and disparities exist by cancer history and occupation. Understanding existing disparities, and the impact of cancer screening on survivors is crucial as the number of working survivors increases.

## Introduction

There is an increasing global cancer burden associated with aging and the world’s population growth, claiming more than 570,000 lives in 2011 in the United States (US) alone. Yet in the past decade the US has seen a significant decline in the diagnoses of advanced stages of the most common cancers (Jemal et al., 2010a). This decrease may in part be attributed to improvements in health behaviors, such smoking, diet, and/or adherence to recommended screening (Jemal et al., 2011). Screening is one of the most important cancer preventive behaviors (Matejic et al., 2011), as such regular screening is likely to have contributed significantly to the reduction of late diagnoses.

Earlier diagnoses and improved treatments have resulted in a growing number of cancer survivors (Jemal et al., 2010b; Lansdorp-Vogelaar et al., 2012) who enjoy better prognoses and a better quality of life. Consequently, survivors are staying or returning to work in greater numbers than before which has two interrelated beneficial consequences. First, survivors, who are more likely to engage in cancer preventive behavior than those with no cancer history (Hudson et al., 2009; Roach et al., 2009), benefit from continued access to employer subsidized health insurance to cover screening costs and, secondly, their experiences and screening behaviors are expected to influence attitudes toward screening within the workplace (Messner and Vera, 2011).

We expect cancer survivors to report higher screening adherence than the general population due to their first-hand knowledge of the benefits of early detection of recurrences and second primary tumors. In fact, 10% of all new cancers are diagnosed in cancer survivors, and these second primaries are the sixth leading cause of cancer deaths (Mayer et al., 2007). However, because employment-based health insurance varies by occupation (Bradley et al., 2002), one may expect differences in adherence to screening, and consequently cancer survivorship by occupation and employment status (Kennedy et al., 2007; National Cancer Institute (NCI), 2007). Our previous work showed health-related disparities by cancer history within working survivors, as well as disparities by occupation (Clarke et al., 2011). Thus, we aim to identify any differences in prevention behaviors by cancer history and occupation. We assessed the adherence to recommended colorectal, breast, cervical, and prostate cancer screening among the general population compared to all cancer survivors and compared to the subpopulation of working survivors.

The specific aim of this study is to evaluate the degree to which the population in general and cancer survivors in particular met recommended screening goals. The collective goals for national screening rates have been set by the US Department of Health and Human Services (USDHHS). These benchmarks are put forward as the “Healthy People objectives” and serve to guide individuals toward making informed health decisions, as well as measure the impact of health-related prevention activities. We used Healthy People 2010 (HP2010) objectives as the benchmark for evaluating whether our populations of interest met cancer screening goals between 1999 and 2010. Throughout this study the term cancer survivor is used to identify persons who have had a cancer diagnosis, are living with the disease, as well as persons who no longer have the disease (National Coalition for Cancer Survivorship (NCCS), 1986).

## Materials and Methods

### Study population

The study population was selected from adult participants (≥18 years) of the National Health Interview Survey (NHIS). The NHIS collects demographic and health information from a representative sample of the non-institutionalized US civilian population annually. Information is collected by household; one adult per family is randomly selected and administered questions related to health, including questions about cancer history and cancer-related health behaviors such as cancer screening.

The study population was divided by cancer status; cancer survivors were then compared to the general population (i.e., persons with and without cancer). We included cancer survivors in the general population because the goals set and screening adherence reported by Healthy People are reflective of all persons regardless of their cancer history. Participants responding “Yes” to “Have you EVER been told by a doctor or other health professional that you had…Cancer or a malignancy of any kind?” were defined as cancer survivors (*n* = 12,990) throughout this study. We included all NHIS records between 1997 and 2010 which provided detailed information on screening behavior. Using the recommendations from the American Cancer Society (ACS) as a guide, we identified individuals ≥18 years who adhered to the recommended screening guidelines for cancers amenable to screening between 1997 and 2010, where data were available (*n* = 174,393).

### Employment status and occupation

Employment status was determined by whether or not respondents were working during the week prior to the NHIS interview. Employed participants were stratified by occupational sector. For occupational sector, we used a four-category variable commonly used by the National Center for Health Statistics, which was based on the 2000 US Census and included the categories of white-collar workers (e.g., banker), service workers (e.g., police officer), farm (including fishing and forestry) workers, and blue-collar workers (e.g., construction worker) (Krieger et al., 2005; Sorerholm, 2006).

### Measuring adherence to screening recommendations

The US Preventive Services Task Force (USPSTF) provides cancer screening recommendations based on comprehensive and systematic reviews of clinical evidence. However the American populace is more familiar with the ACS and the National Cancer Institute (NCI) and their web pages which offer screening information to the public. Thus, the information disseminated by these agencies is likely to influence the screening behaviors of those acting independent of physician recommendations and will reinforce population screening behavior toward meeting the HP2010 goals. In this study we use ACS guidelines to assess the adherence to recommended screening in the US population (Table 1). These guidelines differ slightly from USPSTF, in that ACS recommends an age for first screening mammography 10 years younger than the USPSTF (Smith et al., 2012).

**Table 1:** **Recommendations for Cancer screening as suggested by the American Cancer Society**.

	Screening exam		Recommendations (1999–2010)
Breast cancer screening	Mammography (an digital or film x ray picture of the breast)		Women ≥40 years should have mammograms every 1–2 years
			Women with a higher than average risk of breast cancer should discuss frequency of, and age at first screening with their health care providers
	Clinical breast exam		Every 3 years for women in their 20s and 30s and every year for women ≥40 years
Cervical cancer screening	Pap test*	1999	Sexually active women or those ≥18 years, annual Pap test and pelvic examination. After more than 3 consecutive satisfactory normal annual examinations, the Pap test may be performed less frequently at the discretion of the physician
		2002	At least 3 years after first vaginal intercourse, but no later than 21 years old, women should have regular Pap tests every 1 year or every 2 years with newer liquid-based test. Women ≥30 with 3 consecutive normal Pap test results may get screened every 2–3 years
			Women >30 years may also get screened every 3 years with either the conventional or liquid-based Pap test, in addition to the human papillomavirus (HPV) test
			Women ≥70 years with 3 or more consecutive normal Pap tests and no abnormal Pap test results in the last 10 years may discontinue testing
			Women who have had a total hysterectomy for non-cancer related reasons may discontinue testing
		2009	Screening should begin at age 21
			Women 21–29 years should have Pap test every 3 years. If Pap test result is abnormal then women should have a HPV test
			Women 30–65 years should have both a Pap test and an HPV test every 5 years. It is also okay to have a Pap test alone every 3 years
			Women >65 years who have had regular screenings with normal results should not be screened for cervical cancer
			However those who have been diagnosed with cervical pre-cancer should continue to be screened
Colorectal screening			Men and women ≥50 years
	Flexible sigmoidoscopy		Every 5 years^†^, or
	Colonoscopy		Every 10 years, or
	CT colonography (virtual colonoscopy)		Every 5 years^†^
	Double-contrast barium enema		Every 5 years^†^
	Fecal occult blood test (gFOBT)		Annually^‡^, or
	Fecal immunochemical test (iFOBT/FIT)		Annually^‡^, or
	Stool DNA test (sDNA)		Interval uncertain (possibly 3–5 years)^‡^
Prostate cancer screening	Prostate specific antigen (PSA) blood test/velocity test [How PSA measures rise over time]		Discuss with physician the pros and cons of receiving a baseline PSA and follow-up test if necessary
	PSA density test [Ratio of PSA level to size of prostate gland]		Men at higher than normal risk (Blacks, men whose father, brother or son have been diagnosed with prostate cancer) discuss screening with physician at 45 years
	Percent-free PSA [Ratio of unattached PSA in blood to total PSA]		Men ≥50 years discuss the harms and benefits of PSA screening with physician
	Age-specific PSA range		Men with a previous PSA of ≥4 ng/ml in the blood, should be retested if discussion with physician dictates a necessity
	Digital rectal exam		As recommended by physician

*In 2009 the recommendation for cervical cancer screening changed to pap tests every three years beginning at age 21. Recommendations now include HPV testing every 5 years among women 30–65 years. HPV testing was not included in our analyses. ^†^If test is positive, a colonoscopy should be done. ^‡^A single test done in the doctor’s office is not adequate for testing. A colonoscopy should be done if the test is positive.

Survey participants were asked information regarding relevant screening tests according to their age and gender, regardless of their cancer history. Where appropriate, analyses incorporated changes to recommendations during the course of the study period. For example, historic recommendations for Papanicolaou (Pap) tests were “testing should be initiated among women 18 years of age or among those who are sexually active (whichever is first) once every 2 years if the conventional cervical cells smear was used and every 3 years if the newer liquid-based test was used.” However, in 2009 in accordance with the USPSTF, the ACS changed the recommended age at initial screening to 21 years among women regardless of prior sexual activity, while maintaining the same screening interval. Despite Prostate Specific Antigen (PSA) testing not being endorsed as a general screening test by the USPSTF, we included it in our analyses since prostate cancer is the most commonly diagnosed non-skin cancer and the second leading cause of cancer deaths (after lung cancer) in US men (American Cancer Society (ACS), 2012). We also note that prostate cancer has the highest prevalence rate among US males after non-melanoma skin cancer (Gilligan, 2009).

We measured adherence to screening among the general population and compared them to cancer survivors and working survivors. Using the four occupational sectors (white-collar, blue-collar, farm, and service) we also made comparisons across occupations.

Adherence (outcome of interest) was measured as abiding by any of the recommended guidelines within the specified time frame for gender-specific age groups (Table 1). Persons within the qualifying age and gender categories but whose screening fell outside of the recommended timeframe or who reported not being screened were recorded as non-adherent. In addition to Pap tests and PSA screenings we evaluated adherence to colorectal screening (sigmoidoscopy, colonoscopy, proctoscopy, and home or office stool blood) for men and women, mammogram for women 40 years or older, and manual breast examination for women 20 years or older.

### Healthy people 2010

The HP2010 objectives were set to achieve a 10% improvement in particular health characteristics and screening behaviors within a 10-year period. The baseline was January 2000 figures (the first year of the decade) based on data gathered from the NCHS and other health statistics agencies. The health statistics obtained for the January 2000 benchmarks were obtained from summary measures recorded in 1999.

As Table 1 illustrates, colorectal screening encompasses a variety of screening methods at different intervals. Using the recommendations of the ACS, we examined fecal occult blood test (FOBT), colonoscopy, and sigmoidoscopy jointly. The HP2010 goal was to increase to 50% the proportion of adults aged 50 and older who had had a FOBT within the previous 2 years as well as the proportion of persons in the same age group who had ever had a sigmoidoscopy. There were no separate HP2010 goals for colonoscopy.

Regarding breast cancer screening, the HP2010 goal was to increase to 70% the proportion of women aged 40 and older who had received a mammogram within the previous 2 years. No separate goals were set to address clinical breast exams for 2010. For cervical cancer screening, the goal was to increase to 90% the proportion of women aged 18 and older who had received a Pap test within the previous 3 years (US Department of Health and Human Services (HHS), 2000). Finally, there was no HP2010 target for PSA screening as it is not recommended by any of the governing bodies. The majority of HP2010 goals are consistent with the recommendations used to guide analyses in this study.

### NHIS data

National Health Interview Survey questionnaires did not field all cancer screening questions each year; however, to study changes in preventive cancer screening we analyzed NHIS cancer screening data whenever available within the decade. Data were available for 2000, 2003, 2005, 2008, and 2010. Questions on cancer screening are located in the adult files of 2000, 2003, and 2008 and the periodic cancer module in 2005 and 2010. We used one common method of variable response coding for all data years when the questions of interest were available. For more information about the methods used for cancer screening recodes, see ftp://ftp.cdc.gov/pub/Health_Statistics/NCHS/Program_Code/NHIS/2005/CANCRECO.sas accessed March 12, 2010 and ftp://ftp.cdc.gov/pub/Health_Statistics/NCHS/Program_Code/NHIS/2008/cancreco.sas accessed March 12, 2010. The NCHS uses three different formats for recording information related to reported cancer screening behavior in order to maximize the precision of information obtained as well as the completeness of the data files (Breen et al., 2011). Fundamental differences in formats included the various time codes.

The general questioning across the years included in these analyses was as follows: “When did you have your MOST RECENT (screening exam)?” We examined each format and used complementary but mutually exclusive categories to report adherence to screening guidelines for each year. Participants responses to “date,” “number of time units,” and “time interval” since last screening were re-coded using methods similar to (Breen et al., 2011). The appropriate time interval was chosen for each cancer specific screening according to ACS guidelines. In coding the occupational sectors, The NHIS records information by job type, and industry which can then be classified into the four broad occupational sectors used in this study. The crosswalk for occupation was determined by the notes presented in the Appendix of the adult file and guided by previous studies (Krieger et al., 2005; Sorerholm, 2006).

### Analyses

**Figure 1:**
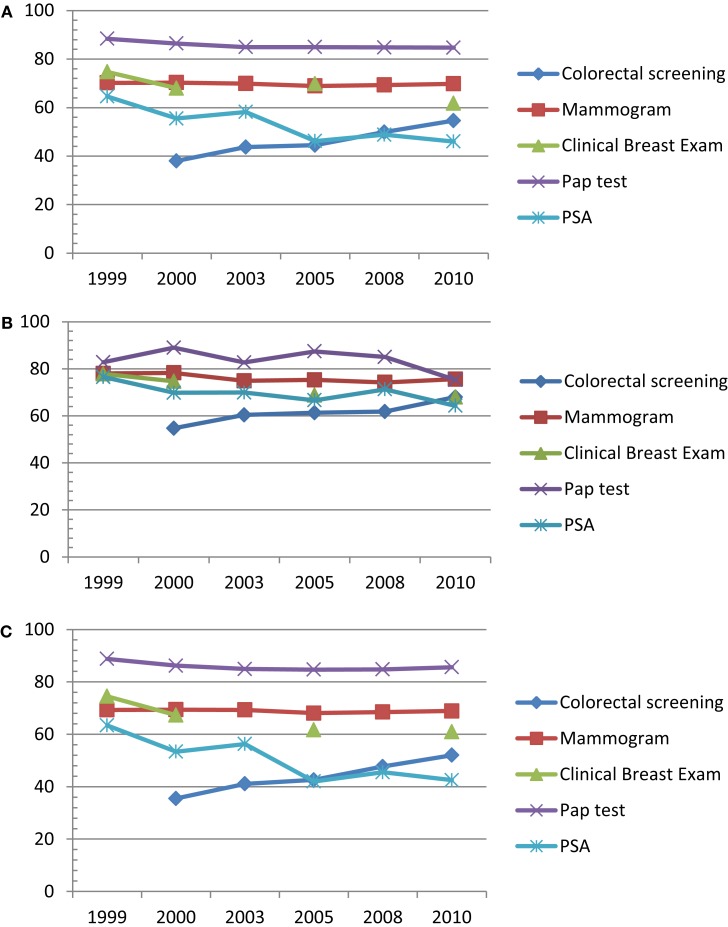
**Trend in adherence to screening among (A) the general US population (*n* = 174,393), (B) US cancer survivors (*n* = 12,990), (C) persons with no prior cancer history (*n* = 161,403)**.

We used methods developed to analyze population-based complex survey data to compute the prevalence of adherence to recommended cancer screening for the general population, as well as for working and non-working cancer survivors over the past decade. For the five screening tests of interest (mammography, clinical breast exam, Pap test, colorectal, and PSA), dichotomous variables were constructed to indicate whether the respondent reported having the test within the recommended time period. The prevalence of cancer specific screening was computed by cancer status, and then by occupational subgroup within the working cancer survivors separately for each year. The SAS survey frequency procedure (SURVEYFREQ) was used to apply the appropriate weights and adjustments for the complex sampling design of the NHIS (SAS Institute Inc, 2003). The line graphs in Figures 1A–C display the resulting screening rates.

To assess for cancer screening trends among working cancer survivors, we used SAS general linear model procedure (GLM) to perform a weighted linear regression of the annual design-adjusted rates for each screening behavior on occupational group and survey year nested in occupational group. This model provides simultaneous estimation of slopes and intercepts for each occupational group. The weight used for each annual rate was the inverse of its design-adjusted variance. We used contrasts to compare the slopes of the regression lines representing each occupation group. We present the estimate of the slope of the regression line, the standard error, and significance level of the slope. We also present comparisons among the slopes of the occupational groups by cancer survivor status. Statistical significance was established at the 0.05 probability level. SAS 9.3 (SAS Institute, Inc., Cary, NC) was used for all analyses.

To create total estimates of US workers (including the subpopulations); we applied the annual sample–person weights and summed them over each annual group and the associated subgroups.

## Results

### Trends among the general population

#### Colorectal cancer screening

Within the general population (cancer survivors included), colorectal screening rates increased by a significant 16.6% representing more than 2.3 million Americans over the past decade (Figure 1A). The most significant change was a 5.7% increase between 2000 and 2003; however, there was an average annual 4.2% change. In 2010 the population met the HP2010 goal of 50% of persons over age 50 years having a colorectal examination with a prevalence of 54.6 ± 1.2% (Table 2).

**Table 2:** **Adherence to recommended cancer screening for most common cancers among **(A)** the general population in the US. *N* = 174, 393. NHIS 1999–2010, **(B)** persons with a prior physician diagnosis of cancer in the US. *N* = 12,990. NHIS 1999–2010, **(C)** persons without a prior physician diagnosis of cancer in the US. *N* = 161,403. NHIS 1999–2010**.

Survey year	1999			2000			2003			2005			2008			2010			HP2010
	Unwt n	Wt%	95% CI	Unwt n	Wt%	95% CI	Unwt n	Wt%	95% CI	Unwt n	Wt%	95% CI	Unwt n	Wt%	95% CI	Unwt n	Wt%	95% CI	
**A**																			
Colorectal screening	N/A	N/A	N/A	4,645	38.0	37.0–39.1	5,292	43.7	42.7–44.8	5,763	44.5	43.3–45.6	4,669	49.9	48.6–51.2	6,407	54.6	53.4–55.8	50%
Mammogram	7,039	70.2	69.2–71.2	6,952	70.3	69.2–71.5	6,968	69.8	68.7–71.0	6,836	68.9	67.8–70.1	5,061	69.3	68.0–70.6	5,980	69.3	68.2–70.6	70%
Clinical breast exam	12,235	74.7	74.0–75.5	8,760	68.0	67.0–69.0	N/A	N/A	N/A	7,971	69.8	68.7–70.9	N/A	N/A	N/A	6,513	61.8	60.6–63.1	None
Pap test	13,543	88.4	87.8–88.9	13,299	86.4	85.6–87.2	12,682	84.7	84.0–85.4	11,032	84.9	84.2–85.7	8,647	84.8	83.9–85.8	9,667	85.8	84.9–86.6	90%
PSA	2,237	64.6	62.7–66.4	1,921	55.5	54.9–58.9	2,086	58.2	56.2–60.3	2,294	46.2	44.5–48.0	1,765	48.8	46.8–50.7	1,978	46.4	44.6–48.2	None
**B**																			
Colorectal screening	N/A	N/A	N/A	901	54.7	52.1–57.4	996	60.4	57.7–63.2	1167	61.3	58.7–63.9	929	61.8	59.3–64.3	638	68.0	65.5–70.5	50%
Mammogram	848	78.0	75.2–80.8	852	78.3	75.7–81.0	819	74.9	72.1–77.7	920	77.4	74.6–80.2	736	74.2	71.0–77.4	887	75.3	72.7–77.8	70%
Clinical breast exam	951	77.8	75.3–80.4	887	74.7	71.8–77.6	N/A	N/A	N/A	901	68.8	66.1–71.4	N/A	N/A	N/A	1270	68.0	65.5–70.5	None
Pap test	934	82.8	80.4–85.1	994	89.0	86.7–91.3	904	82.7	80.1–85.3	873	87.4	85.3–89.5	726	85.0	82.3–87.6	839	77.8	75.2–80.4	90%
PSA	442	75.6	71.7–79.4	353	67.4	63.1–71.7	414	67.9	65.4–74.2	501	65.6	61.7–69.5	356	69.1	65.3–72.9	445	64.0	60.7–67.3	None
**C**																			
Colorectal screening	N/A	N/A	N/A	3,743	35.5	34.4–36.6	4,288	41.1	40–42.3	4,592	42.6	40.5–42.8	3,735	47.7	46.3–49.2	5,136	52.0	50.8–53.3	50%
Mammogram	6,186	69.3	68.2–70.4	6,099	69.4	68.1–70.6	6,143	69.3	68.1–70.5	5,929	68.1	66.9–69.4	4,317	68.5	67.1–69.9	5,092	68.5	67.2–69.7	70%
Clinical breast exam	11,275	74.5	73.7–75.3	7,871	67.3	66.3–68.4	N/A	N/A	N/A	6,973	61.7	60.5–62.8	N/A	N/A	N/A	5,689	61.0	59.6–62.3	None
Pap test	12,380	88.8	88.2–89.4	12,291	86.2	85.4–87.0	11,767	84.9	84.2–85.6	10,152	84.7	83.9–85.5	7,798	84.8	83.9–85.8	7,951	86.6	85.7–87.5	90%
PSA	1,794	63.4	61.4–65.4	1,566	53.4	51.4–65.5	1,669	56.3	54.2–58.5	1,793	42.0	40.2–43.7	1,408	45.5	43.3–47.2	1,522	42.5	40.6–44.3	None

#### Breast cancer screening

Rates for mammography showed little change between 1999 and 2010, with an average reported adherence of 69.7%. The US approached but failed to meet the HP2010 goal of 70% of eligible women having received a mammogram in the previous 2 years in 2010. The reported proportion was at 69.4% representing more than 3.3 million women over the age of 40 years. There was an average annual change of −4.3% and a prevalence of 61.8 ± 1.2% representing more than 3.7 million women in 2010 for CBEs.

#### Cervical cancer screening

There was a 3.7% decrease in self-reported Pap test among women 18 years and older between 1999 and 2010. The HP2010 goal for cervical cancer screening in women above 18 years was 90%. When screening adherence was defined based on the recommendations issued earlier in the decade, 84.7% of the population in 2010 reported adherence to Pap test guidelines. However, there was a slight increase to 85.5% in the same population when adherence included the change in recommendations in 2009.

#### Prostate cancer screening

In 1999, 64.6% of the eligible male population reported getting a PSA test in the previous 12 months. There was an average annual decline of −3.6% until 2010 with a reported prevalence of 46.0%representing an estimated 1.3 million men over 50 years.

### Trends among cancer survivors

Overall, Cancer survivors (Figure 1B) demonstrated a comparatively higher adherence to recommended screening than the persons with no cancer history.

#### Colorectal cancer screening

There was a lower average annual increase in colorectal screening (3.3%; 18,554 persons) compared to the general population, but baseline screening rates were on average approximately 10% higher. Survivors were consistently above the HP2010 colorectal screening goal of 50% throughout the entire decade.

Series of trend graphs illustrating adherence to cancers amenable to routine screening, according to ACS guidelines. Data source: NHIS 1997–2010.

#### Breast cancer screening

Among survivors, mammography declined between 2000 and 2003 (78.3 ± 2.6% to 74.9 ± 2.8%) and again between 2005 and 2008 (77.4 ± 2.8% to 74.2 ± 3.2%), but increased to 75.6% in 2010 (Table 2). Despite the decade long fluctuation in screening prevalence, cancer survivors surpassed the HP2010 goal for mammography screening. An estimated 488,104 women reported receiving a mammogram within the previous 2 years in 2010. Reported CBEs declined among survivors from 77.8 ± 2.5% in 1999 to 68.0 ± 2.5% in 2010. The average annual decline of 3.2% over the decade represented an estimated 403,084 eligible women who reported not having a clinical breast examination in the recommended period. No CBE screening data were available for 2003 and 2008.

#### Cervical cancer screening

There was a small annual average decline of 1.5%, representing an approximate 38,000 fewer women who reported receiving a recommended pap test between 1999 and 2010. There were undulations in the trend in adherence throughout the decade. This is the only cancer for which survivors failed to meet the HP2010 goal throughout the decade.

#### Prostate cancer screening

Compared to the general population, survivors received PSA testing at a consistently 10–20% higher rate. Within this group the trend across the years showed a decline from 76.5 ± 4.1% in 1999 to 64.3 ± 2.9% in 2010.

### Trends among persons without a history of cancer

Persons without a history of cancer demonstrated lower adherence to recommended screening guidelines for most cancers amenable to screening compared to cancer survivors (Figure 1C).

#### Colorectal cancer screening

The average annual increase in colorectal screening was 4.1% representing an estimated 473,573 more persons screened each year. Persons without a history of cancer surpassed the HP2010 goal for colorectal screening in 2010 at 52.0%.

#### Breast cancer screening

Mammography trend was similar to the general population. There was a decline between 2000 and 2005 (69.4 ± 1.3% to 68.1 ± 1.2%), followed by an increase to 68.5% in 2010. Reported CBEs declined from 74.5 ± 0.8%in 1999 to 61.0 ± 0.4% in 2010, an estimated decrease of 654,130 examinations during the recommended period (Table 2).

#### Cervical cancer screening

Though there was a small annual average decline of 0.9%, a cumulative 2.5 million fewer women reported receiving a recommended pap test between 1999 and 2010. The population failed to meet the HP2010 goal throughout the decade.

#### Prostate cancer screening

Among men without a history of cancer, the trend in PSA testing declined from 63.4 ± 2.0% in 1999 to 42.5 ± 1.9% in 2010. This represents more than 5.5 million men over 50 years old who did not participate in PSA screening since 1999.

### Trends among working survivors

Disparities in screening across occupational sectors were observed over the decade among working cancer survivors (*n* = 7,528). Farming sector estimates were not presented because of small sample sizes. Table 3 shows that the annual cancer screening rates among working cancer survivors significantly increased for only four of 15 cancer screening/occupation subgroups over the period 1999–2010. The percent change is a summary measure of the difference in prevalence of adherent screening between 1999 and 2010. Table 5 illustrates the results of comparing the trend (slope) in screening over the decade.

**Table 3:** **Trends in Recommended screening among working cancer survivors (National health interview survey 1999–2010)**.

Survey year	Average annual sample size		Percent adherent to screening						Average annual% change	Regression	
	Observed N	Population estimate	1999	2000	2003	2005	2008	2010		Slope ± SE^b^	*P*^c^
**COLORECTAL SCREENING^a^**											
White-collar	480	275,468	n/a	51.3	66.6	67.3	66.0	71.8	5.2	0.014 ± 0.003	< 0.001
Blue-collar	150	83,840	n/a	32.1	47.2	59.4	58.7	63.6	7.9	0.018 ± 0.021	0.004
Service	94	50,454	n/a	48.9	62.0	54.8	57.4	62.3	3.4	0.011 ± 0.006	0.075
**MAMMOGRAM^a^**											
White-collar	388	208,109	84.1	81.2	78.8	81.7	80.5	77.4	−1.3	−0.003 ± 0.002	0.230
Blue-collar	60	30,142	63.9	58.1	77.1	69.9	68.5	76.3	2.5	0.013 ± 0.006	0.024
Service	268	36,162	88.4	84.0	82.5	64.5	59.3	72.1	−3.3	−0.005 ± 0.006	0.461
**CLINICAL BREAST EXAM^a^**											
White-collar	419	188,170	88.5	83.0	n/a	75.2	n/a	64.8	−7.9	−0.117 ± 0.002	<0.001
Blue-collar	68	330,507	78.4	63.3	n/a	63.2	n/a	64.0	−4.8	−0.007 ± 0.005	0.201
Service	76	35,832	87.3	71.3	n/a	58.3	n/a	62.6	−8.2	−0.003 ± 0.006	0.662
**PAP TEST^a^**											
White-collar	415	221,681	91.7	93.6	89.6	88.8	88.0	79.3	−4.7	−0.007 ± 0.002	<0.001
Blue-collar	66	32,245	87.6	93.7	92.6	79.5	76.1	70.1	−4.6	−0.011 ± 0.004	0.01
Service	84	44,096	89.2	96.9	89.4	88.6	80.2	84.0	−2.0	−0.006 ± 0.004	0.18
**PROSTATE SPECIFIC ANTIGEN TEST^a^**											
White-collar	152	96,002	78.4	70.0	69.0	71.5	74.8	66.8	−2.3	−0.002 ± 0.004	0.613
Blue-collar	78	46,138	69.8	52.5	81.0	61.3	62.3	59.7	−2.0	−0.006 ± 0.008	0.437
Service	32	17,302	73.2	64.5	61.9	49.7	71.5	64.4	−1.8	0.009 ± 0.007	0.247

*^a^Too few observations in farming sector*.

*^b^Standard error of the mean*.

*^c^*p*-value*.

#### Colorectal cancer screening

Based on the average population, the increase among working survivors white-collar (5.1%), blue-collar (7.9%), and service workers (3.4%) who received colorectal screening is representative of an estimated 70,245; 33,117, and 8,830 Americans within each sector, who reported following recommended guidelines. There were significant differences in colorectal screening trends over the decade between white-collar and blue-collar working survivors (*p* = 0.016).

#### Breast cancer screening

There was an average annual 1.3% decline among white-collar workers representing 16,233fewer women who reported not receiving mammograms in between 1999 and 2010. There was an even larger average annual decline (−3.3%) in the service sector, but an average increase of 2.5% among blue-collar workers. There was a decline in CBEs between 1999 and 2010 across all sectors with the largest average change occurring in the service sector (−8.2%) and the smallest change within the blue-collar sector (−4.8%). There were no significant within group differences in mammography trends among working survivors, however the trend in CBE was significantly different between white-collar and blue-collar working survivors (*p* = 0.005).

#### Cervical cancer screening

There was a decrease in adherence to Pap test recommendations across all occupation sectors between 1999 and 2010; except for service workers who demonstrated a 3.8% increase in adherence in 2010 when compared to 2008. In 1999, survivors employed in the white-collar sector met the 2010 goal at 92.9%. They maintained a greater than 90% of population screened until 2003, after which the continued decline led to all workers falling below recommended screening levels. The adherent population within all three sectors was between 8 and 20% below the HP2010 goal in 2010. White-collar survivors showed a significant difference in the trend in Pap tests over the decade when compared to working survivors in blue-collar (*p ≤ *0.0001) and service <0.0001) occupations. There were no significant differences in trends between service and blue-collar working survivors (Table 5).

#### Prostate cancer screening

An estimated average 158,850 male working cancer survivors over the age of 50 years reported having a PSA test within 1 year of the NHIS interview. All occupational sectors were characterized by a greater than 70% adherence to annual PSA testing guidelines among working male survivors in this age group. Whereas there was an average decline between 1999 and 2010 within all occupations, there were fluctuations in trends across time. There were no significant within group differences in PSA screening trends among working survivors.

### Trends among workers with no history of cancer

#### Colorectal cancer screening

Based on the average population, there was an estimated increase of 313,886 white-collar workers, 100,923 blue-collar workers and 64,371 service workers who reported having a colorectal screening examination within the recommended time frame. The average yearly increase in screening showed little variation between occupational groups (Table 4). Comparisons of trends in colorectal screening between working persons without a cancer history yielded no significant differences (Table 5).

**Table 4:** **Trends in recommended screening among working persons with no cancer diagnosis (National health interview survey 1999–2010)**.

Survey year	Average annual sample size			Percent adherent to screening						Average annual% change	Regression	
	Observed *N*	Population estimate		1999	2000	2003	2005	2008	2010		Slope ± SE^b^	*P*^c^
**COLORECTAL SCREENING^a^**												
White-collar	1,998	1,162,541		n/a	36.7	42.3	48.5	53.9	58.2	5.4	0.023 ± 0.001	<0.0001
Blue-collar	652	366,995		n/a	24.8	34.1	37.7	44.0	46.8	5.5	0.022 ± 0.002	<0.0001
Service	470	242,911		n/a	25.8	32.5	35.7	41.9	47.0	5.3	0.023 ± 0.002	<0.0001
**MAMMOGRAM^a^**												
White-collar	2,812	1,490,118		76.4	74.3	74.8	73.9	72.8	73.6	−0.6	0.003 ± 0.001	0.669
Blue-collar	493	221,237		64.1	59.9	63.9	62.1	61.0	60.1	−0.8	−0.001 ± 0.002	0.698
Service	567	275,723		60.4	66.6	57.8	60.7	64.5	62.4	0.4	0.005 ± 0.002	0.009
**CLINICAL BREAST EXAM^a^**												
White-collar	4,303	2,098,990		82.1	76.6	n/a	69.1	n/a	67.2	−5.0	−0.010 ± 0.001	<0.001
Blue-collar	649	306,093		72.4	63.2	n/a	50.4	n/a	49.1	−7.8	−0.019 ± 0.002	<0.001
Service	768	347,708		68.9	0.6	n/a	54.5	n/a	55.0	−4.6	−0.005 ± 0.002	<0.001
**PAP TEST^a^**												
White-collar	5,560	2,917,742		92.9	90.9	90.4	89.3	87.9	88.4	−1.8	−0.002 ± 0.0004	<0.001
Blue-collar	1,025	457,033		88.0	83.7	83.3	78.2	80.4	81.5	−2.9	−0.003 ± 0.001	0.005
Service	1,182	598,622		87.2	83.4	82.5	81.1	84.1	85.0	−1.4	0.003 ± 0.001	0.682
**PROSTATE SPECIFIC ANTIGEN TEST^a^**												
White-collar	639	394,574		60.7	61.4	56.0	49.0	51.6	49.8	−2.2	−0.005 ± 0.002	0.003
Blue-collar	308	186,188		52.9	48.5	55.3	37.3	41.7	36.5	−3.3	−0.004 ± 0.003	0.012
Service	177	95,329		55.8	43.3	49.8	35.4	39.3	33.9	−4.4	−0.014 ± 0.003	<0.001

*^a^Too few observations in farming sector*.

*^b^Standard error of the mean*.

*^c^*p*-value*.

**Table 5:** **Comparison of trend (slopes) in adherence to cancer screening over the decade (National health interview survey 1999–2010)**.

	Colorectal screening	Mammogram	Clinical breast exam	Pap test	PSA
	Est^a^ ± SE^b^	Est. ± SE	Est. ± SE^b^	Est. ± SE^b^	Est. ± SE^b^
**GENERAL POPULATION**					
Survivor vs. no cancer history	0.019 ± 0.016 *p*^c^ = 0.242	0.028 ± 0.024 *p* = 0.252	0.034 ± 0.025 *p* = 0.167	0.030 ± 0.017 *p* = 0.069	0.059 ± 0.019 *p* = 0.002
**WORKING CANCER SURVIVORS**					
WC^d^ vs. BC^e^	0.008 ± 0.003 *p* = 0.016	0.002 ± 0.003 *p* = 0.374	0.007 ± 0.003 *p* = 0.005	0.010 ± 0.002 *p* ≤ 0.0001	0.006 ± 0.005 *p* = 0.217
WC vs. service	0.006 ± 0.006 *p* = 0.339	−0.008 ± 0.006 *p* = 0.200	−0.002 ± 0.005 *p* = 0.748	0.022 ± 0.005 *p* ≤ 0.0001	0.005 ± 0.008 *p* = 0.554
BC vs. service	0.077 ± 0.021 *p* = 0.726	−0.037 ± 0.031 *p* = 0.242	−0.032 ± 0.032 *p* = 0.320	−0.028 ± 0.022 *p* = 0.191	−0.042 ± 0.024 *p* = 0.073
**WORKING PERSONS WITHOUT A HISTORY OF CANCER**					
WC vs. BC	0.002 ± 0.021 *p* = 0.908	0.022 ± 0.032 *p* = 0.486	−0.001 ± 0.033 *p* = 0.968	0.038 ± 0.022 *p* = 0.079	0.016 ± 0.023 *p* = 0.482
WC vs. service	0.004 ± 0.009 *p* = 0.633	0.004 ± 0.010 *p* = 0.703	−0.015 ± 0.009 *p* = 0.103	0.013 ± 0.007 *p* = 0.067	−0.017 ± 0.011 *p* = 0.131
BC vs. service	0.002 ± 0.020 *p* = 0.923	−0.018 ± 0.031 *p* = 0.560	−0.013 ± 0.032 *p* = 0.682	−0.025 ± 0.022 *p* = 0.246	−0.033 ± 0.023 *p* = 0.152

*^a^Estimate*.

*^b^Standard error*.

*^c^*p*-value*.

*^d^White-collar*.

*^e^Blue-collar*.

#### Breast cancer screening

There was an average annual 0.6% decline among white-collar workers representing 53,644 fewer women who reported not receiving mammograms in 2010 when compared to their 1999 cohort. There was a larger average annual decline (−0.8%) in the blue-collar sector, but an average increase of 0.4% among service workers. There was a decline in CBEs between 1999 and 2010 across all sectors with the largest average change occurring in the blue-collar sector (−7.8%) and the smallest change within the service sector (−4.6%). The reported decline in proportion of CBEs represented more than 95,000 and 63,000 eligible blue-collar and service workers who failed to seek screening within the recommended time frame each year throughout the decade. Between occupations investigations of trends in breast cancer screening among working persons without a cancer history showed no significant differences.

#### Cervical cancer screening

There was a decrease in adherence to Pap test recommendations across all occupation sectors. The decline was consistent within the white-collar and blue-collar occupation sectors until 2008 after which there was an increase of approximately 1.0% in 2010. The service sector workers demonstrated an earlier elevation in adherence with a 3.0% increase between 2000 and 2008 and an approximate 1.0% increase between 2008 and 2010. With the annual average population estimated at more than 2.9 million, white-collar workers met the HP2010 goal between 1999 and 2003. No other sector met this goal throughout the decade. There were no significant differences in trends in cervical cancer screening between occupation groups among working women without a cancer history.

#### Prostate cancer screening

Since 1999, more than 552,000 working men over the age of 50 years refrained from having an annual PSA test. PSA screening was much lower among this population when compared to working survivors. There was an average decline between 1999 and 2010 within all occupations, with the largest observed decline among the service sector (−4.4%). This was twice the rate of decline among white-collar workers and a third higher than blue-collar workers. In 2010 an average 40.1% of this subpopulation reported having a PSA test in the past 12 months. They represented 270,437 of working men over 50 years of age without a cancer history. There were no significant differences in PSA screening trends between occupation groups among working persons without a history of cancer.

## Discussion

Although cancer-related mortality has declined in part due to early detection, there has been continued debate regarding the adequacy of screening and the over-diagnosis of indolent cancers (Mandelblatt and Cronin, 2011). Our results indicate that there has been a general decline in adherence to recommended screening throughout the decade, and that the general US population failed to meet the HP2010 goal for all targeted screening exams except colorectal screening. Cancer survivors, a population with an increased risk of developing recurrences or new primary tumors, met the goals for all common screening exams except cervical cancer. However this group also illustrated a downward trend for most screenings in the last 3 years of the study period. This declining trend foreshadows a future negative impact on mortality from cancers of the breast, and cervix as well as increased morbidity associated with a later diagnosis of prostate cancer. Disagreements among the USPSTF, the ACS and other recommending bodies over cancer screening guidelines may have contributed to the decline in screening throughout the decade (Gotzsche, 2005; Kaplan, 2009; Hendrick and Helvie, 2011; Takahashi et al., 2011). A decline in worker insurance rates over the decade under study (Cunningham et al., 2008; McCollister et al., 2010) could also be a contributing factor.

### Breast cancer trends reflect uncertainty in screening

Whereas the average decline in mammography screening was low, it still translated into over 31,000 fewer women reported being screened in the previous 2 years in 2005 as compared to 2003. The general population failed to meet the HP2010 goal of 70% for most of the decade, except in 2010 when it was met. Survivors exceeded the mammography goal throughout the entire decade but showed a larger decline between 2000 and 2005 than the general population. The difference in trend between these groups was significant. Others reported that the greatest decline in mammograms had been among women between 50 and 59 years of age as well as among highly educated women in white-collar jobs (Breen et al., 2007). Breast cancer screening prevents over 2.0% of cancer deaths annually (Altekruse et al., 2010), thus early detection is of key importance to the groups at increased risk such as breast cancer survivors (Brewster et al., 2008) and women over 50 years of age (Mandelblatt et al., 2009). We did not detect any significant occupational differences in the rate of change in breast cancer screening within groups according to cancer history.

### Increasing HPV vaccination may contribute to decreasing pap tests

We observed a higher cervical screening rate when we examined women 21 years and older compared to women 18 years and older. On average, approximately 131,000 fewer women had a pap test each year, with a significant decline between 2003 and 2005. There was a declining trend among cancer survivors, but the most noticeable decrease occurred after 2008. Though not explored in our analyses due to data limitations, we speculate that in addition to changing guidelines this decline may be associated with the extension of the Human Papillomavirus (HPV) vaccine to young women up to 26 years. There has been a 10.2% increase in HPV vaccination among women 19–26 years since its introduction to this age group in 2008 (Williams, 2012). However, as the available vaccines protect women from only two to four of the many cancer-causing HPV infections, this trend of higher vaccinations at the price of lower screening may be more detrimental than the previous unavailability of a vaccine but higher screening rates (Harper, 2009). Unexpectedly, cancer survivors had a lower screening rate than their peers without a prior diagnosis for several periods over the decade but there was no significant difference in trend between the two groups, overall. This important decline underlines the urgent need of implementing information campaigns emphasizing the need to continue screening despite the availability of an effective but limited vaccine.

### PSA screening declined as its effectiveness was questioned

In 1999, 1.1 million men participated in PSA screening within a 12-month period. They represented 64.6% of males over 50 years of age. This proportion decreased throughout the decade but in 2010 NHIS data reflected more than1.3 million men who had a PSA test within a 12-month period. Though population estimates pointed to an increase in the number screened, this proportion was representative of 46.0% of males over 50 years, an almost 20% decline since 1999. Based on recent reports on the deficiencies of PSA as a diagnostic tool (Thompson et al., 2006; Simmons et al., 2011), the consensus of all recommending bodies is that the risk of routine PSA screening (e.g., unnecessary tests and/or surgeries for indolent cancers) far outweighs the benefits. This and other reports of the greater likelihood of outliving the disease among most men may have influenced the observed decline. Though not as large as among the general population, there was an observed reduction in the number of male cancer survivors who reported having an annual PSA screening test. The trends for these two groups were significantly different.

### HP2010 goals met for colorectal screening

Based on a consistent increase since 2003, the US population achieved and maintained the HP2010 goal of 50% adherence to ACS guidelines for colorectal screening from 2008 through 2010. Cancer survivors, who already started out at 54.7% at the beginning of the decade, experienced a smaller increase between 2008 and 2010. Routine screening can reduce the number of people who die from colorectal cancer by at least 60% (Center for Disease Control and Prevention (CDC), 2012), and though increasing, the proportion screened is still disproportionate to the proven benefits of early detection.

### Level of adherence by cancer history

There are a myriad of determinants affecting a person’s decision to seek cancer screening such as perceived vulnerability to cancer (Calvocoressi et al., 2004; Rutten et al., 2005) or level of acceptability of screening practices (Breen et al., 2011). Whereas screening rates for some cancers are still below recommended levels, cancer survivors consistently exceeded population rates by over an average 10% throughout the decade. Survivors exceeded the HP2010 goal in spite of a decline in rates since 2000. A small but significant downward trend was observed in the proportion of women who reported having had a Pap test within the previous 3 years, regardless of cancer history. However, rates were consistently higher among survivors. It is assumed that survivors are more likely to adhere to recommended cancer screening due to their experience with the disease, awareness of their increased risk for second cancers, and first-hand knowledge of the benefits of screening. However, the experience of continuing working or returning to work varies for survivors according to occupation, e.g., access to health insurance coverage, which may influence adherence to screening. As such, employment disparities among survivors must be addressed (Messner and Vera, 2011).

### Screening differs among working survivors

In general, the more highly educated, white-collar cancer survivor employed in an occupation which provides comprehensive private health insurance is more likely to be among the early adopters of screening practices (Fleming et al., 2007). We found that white-collar workers had significantly higher screening rates of mammography, CBEs, and pap tests compared to blue-collar workers, but were comparable to service workers. However, since 2005 breast cancer screening among white-collar workers declined while increasing among blue-collar and service workers. Previous occupational health studies identified blue-collar workers as the working population most likely to report poor health, functional limitations, lack of information on preventive health behaviors and reduced healthcare access and utilization (Quintiliani et al., 2007; Clarke et al., 2011). Disparate health behaviors among this worker group are often magnified by chronic diseases such as cancer. Historically, blue-collar workers have reported taking fewer days-off and sick days than their white-collar peers regardless of reported health status (Lee et al., 2006).

White-collar survivors, similar to their colleagues without a cancer diagnosis, appeared to be more receptive to cancer screening than survivors employed in the service and blue-collar sectors. Service workers do not fare much worse than white-collar workers with regards to many known health benefits (Schmitt, 2009), and did not fall far behind in adherence to screening, especially for Pap tests and PSA screening. Public debate over the value of cancer screening will continue as new scientific discoveries lead to changing guidelines. This may or may not translate into reduced adherence to screening. In fact, our results showed that the US achieved the HP2010 goal of 50% adherence to recommended colorectal screening and 70% adherence to mammography screening despite the change in recommendations along the decade. On the other hand, women 18 years and older experienced a decline in pap tests over the past few years and the HP2010 goal remained unmet, and mammography screening fluctuated over the decade. Whether the new mammography and Pap test guidelines will influence future screening decisions is uncertain. It is also unknown if the USPSTF’s reiteration of the ineffectiveness of PSA as a population-wide cancer screening tool will lead to further reductions in testing. Physicians continue to recommend PSA screening for high risk patients but existing barriers such as those identified by Pollack et al. (2012), may be further complicated by reinforcing the message of more harm than good with regards to this screening exam. Despite demonstrating higher screening rates than the general population, it is evident that cancer survivors had a slight decline in screening rates and are in need of continued medical advice post-treatment and diagnosis. Further, screening rates among survivors differ according to occupational sectors.

Our findings should be interpreted in the context of the study’s limitations. First, the data for our main outcome variables, screening procedures, are self-reported. However, our primary goal was to analyze trends over time, and the expected rate of over-reporting of a “desirable behavior” may be assumed to not vary significantly across survey years. As a result, the bias introduced is unlikely to impact our main findings significantly and, thus, we assume that our results regarding percent change in screening behaviors reflect actual trends. Second, the sample size of cancer survivors employed in the farming sector, was too small for analysis, thus, our comparisons by occupational sector are limited to white-collar, blue-collar, and service workers.

Finally, due to data limitations, we do not know what type of Pap test (liquid-based or glass smear) was performed on women screened for cervical cancer. Because the time between screenings varies from 2 to 3 years depending on the test used, we may have under- or over-estimated those rates. Further, we had no data on the age at first coitus which may determine the recommended time of the first Pap test (at 18 years of age or 3 years after first sexual activity).

Our research findings are strengthened by the use of the NHIS, a nationally representative sample of the entire US population, which yielded a pooled sample of more than 7,500 working cancer survivors available for analysis. Consequently, the data reflect similar screening rates in the general population presented by the NCI in earlier years for some of the cancers assessed in this research (National Cancer Institute (NCI), 2010). The use of a trend analysis to investigate changes provides a systematic review of historical patterns in screening behavior while permitting the assessment of occupational differences within each year.

In summary, population-based information about cancer survivors is useful for researchers, public health practitioners, and program implementers responsible for the development and assessment of interventions aimed at improving the health and quality of life in this growing segment of the US population. Examining this information by occupational group is the next logical step in the effort to overcome any existing structural inequalities existing within working cancer survivors and promote policies aimed at meeting the needs of all employed groups (Dani and De Haan, 2008). However, this is a difficult task as most laws and public policies are written in a general format. Unfortunately, a “one-size-fits-all” approach will not lead to a decrease in occupation-related disparities in cancer screening. Hence, we hope that further research in this field will also examine the combined effect of other social determinants (psychosocial, logistic, and financial) that may influence screening decisions. A comprehensive approach may better inform the design of targeted workplace interventions to increase screening among the general population, with a special focus on cancer survivors, and tailored to the challenges and opportunities afforded within each occupational sector.

## Conflict of Interest Statement

The authors declare that the research was conducted in the absence of any commercial or financial relationships that could be construed as a potential conflict of interest.
